# Exogenous Magnesium Application as a Salinity Mitigator in Cashew Genotypes

**DOI:** 10.3390/plants15010037

**Published:** 2025-12-22

**Authors:** Alexandre Xavier de Oliveira, Paulo Cássio Alves Linhares, Gabriel Sidharta dos Santos Rego, Rita de Cássia do Nascimento Medeiros-Sá, Luan Cordeiro de Souza Barbosa, Janildo Pereira da Silva Júnior, Diogo Santos Cavalcante, Alex Alvares da Silva, Edivan da Silva Nunes Júnior, Kleane Targino Oliveira Pereira, Miguel Ferreira Neto, Salvador Barros Torres, Tayd Dayvison Custódio Peixoto, Alberto Soares de Melo, Francisco Vanies da Silva Sá

**Affiliations:** 1 Department of Agrarian and Exact, Universidade Estadual da Paraíba, Catolé do Rocha 58884-000, PB, Brazil; alexandre.xavier@aluno.uepb.edu.br (A.X.d.O.); paulocassio@servidor.uepb.edu.br (P.C.A.L.); gabriel.rego@aluno.uepb.edu.br (G.S.d.S.R.); rita.medeiros@aluno.uepb.edu.br (R.d.C.d.N.M.-S.); luan.barbosa@aluno.uepb.edu.br (L.C.d.S.B.); janildo.junior@aluno.uepb.edu.br (J.P.d.S.J.); diogo.cavalcante@aluno.uepb.edu.br (D.S.C.); alex.alvares@visitante.uepb.edu.br (A.A.d.S.); edivanuepb@servidor.uepb.edu.br (E.d.S.N.J.); 2 Department of Agronomic and Forest Sciences, Universidade Federal Rural do Semi-Árido, Mossoró 59625-900, RN, Brazil; kleane.pereira@alunos.ufersa.edu.br (K.T.O.P.); miguel@ufersa.edu.br (M.F.N.); sbtorres@ufersa.edu.br (S.B.T.); 3 Center of Agrarian and Biological Sciences, Universidade Estadual Vale do Acaraú, São Benedito 62370-000, CE, Brazil; tayd_custodio@uvanet.br; 4 Department of Biological Sciences, Universidade Estadual da Paraíba, Campina Grande 58429-900, PB, Brazil; alberto.melo@servidor.uepb.edu.br

**Keywords:** Anacardiaceae, salinity, foliar nutrition

## Abstract

Cashew (*Anacardium occidentale* L.), native to northeastern Brazil, holds significant socioeconomic value, but its cultivation is limited by salinity, which is common in semiarid regions. This study evaluates foliar magnesium (Mg) application as a strategy to mitigate salinity stress in cashew seedlings. A greenhouse experiment was conducted with two genotypes (CCP 76 and AT01), two irrigation salinity levels (0.5 and 2.5 dS m^−1^), and three Mg doses (0, 1, and 2 mL L^−1^). Salinity reduced growth, physiological parameters, and stomatal conductance. Foliar Mg application, particularly at 1 mL L^−1^, alleviated these effects by increasing root dry mass, stomatal conductance, internal CO_2_ concentration, and intrinsic water-use efficiency, especially in genotype AT01. The 2 mL L^−1^ dose showed inconsistent responses, suggesting toxicity. Overall, Mg application mitigates salinity effects in cashew, with efficiency dependent on genotype and dose, and AT01 demonstrating greater tolerance.

## 1. Introduction

The increase in the electrical conductivity of irrigation water can cause significant agricultural yield losses. According to the Food and Agriculture Organization of the United Nations [1], the global economic cost associated with salt-induced soil degradation in irrigated areas is estimated at US$27.3 billion annually. Reductions in crop growth and productivity occur as a response to osmotic and ionic stress.

The osmotic effect arises from the high concentration of salts in the root zone, which reduces the osmotic potential of the soil and, consequently, water availability to plants [2]. The ionic effect, in turn, is related to the accumulation of ions such as Na^+^ and Cl^−^, which can be toxic to plants [3]. This ion accumulation also causes nutritional imbalances, altering the uptake, transport, and distribution of essential nutrients. For instance, excess Na^+^ may inhibit the absorption of potassium (K^+^), calcium (Ca^2+^), and magnesium (Mg^2+^), while chloride (Cl^−^) may hinder nitrate (NO_3_^−^) and dihydrogen phosphate (H_2_PO_4_^−^) uptake [4,5,6]. Identifying varieties that tolerate the adverse effects of salinity at the seedling stage may therefore facilitate crop establishment in the field.

Cashew (*Anacardium occidentale* L.) exhibits wide genetic variability, notably between the common and dwarf cashew groups. This diversity results from a long process of natural selection and domestication, which enabled the species to adapt to diverse environmental conditions. In Northeastern Brazil, where cashew is native, this genetic variability has been exploited to develop cultivars adapted to local conditions, thereby enhancing the socioeconomic importance of this crop [7,8,9]. In 2023, cashew nut production in Brazil reached 127,931 tons, totaling R$453.163 million; however, output declined by 12.7% compared with 2022 [10].

Seedling production is a critical stage for agricultural success, as seedling quality is directly linked to the productive potential of field-grown plants. Nonetheless, Northeastern Brazil faces major challenges due to its predominantly semi-arid climate, where water availability is a serious concern. This scarcity is compounded both by insufficient quantity, with evapotranspiration exceeding precipitation, and by poor quality, since high salt concentrations are present in local wells, reservoirs, and rivers [11].

The AT01 cashew clone, a precocious dwarf genotype, has stood out in Brazilian cashew production due to its high yield and fruit quality [12]. Similarly, CCP 76, a precocious dwarf clone developed by Embrapa, is widely cultivated in commercial orchards, largely due to its high productivity and earliness. Carneiro et al. [9] evaluated the sensitivity of CCP 76 to saline stress during pre-flowering, reporting that this genotype is “moderately sensitive” to irrigation water salinity in this phase. An electrical conductivity of irrigation water (ECw) = 1.6 dS m^−1^ was considered the salinity tolerance threshold for pre-flowering in this cashew genotype.

The use of moderately saline water for irrigation may be feasible if the maximum salt concentration tolerated by plants without yield losses is known. However, it is essential to determine the tolerance capacity of each crop, as genotypes differ, and to identify strategies that mitigate the negative effects of salinity.

Magnesium, essential for photosynthesis and other vital processes, has been little investigated in the context of salinity tolerance despite its crucial role in plant productivity [13]. Although considered a secondary macronutrient, magnesium is indispensable for several physiological and biochemical processes, including chlorophyll synthesis and degradation and the activation of multiple antioxidant enzymes [14]. It also plays a role in carbohydrate metabolism and in the activation of enzymes related to energy metabolism, both essential for plant growth and development [15,16,17]. We hypothesize that exogenous application of magnesium can mitigate salt stress in cashew genotypes with different degrees of salinity tolerance. In this context, the present study aimed to evaluate magnesium application as a mitigator of salt stress in cashew seedlings.

## 2. Results

**Figure 1:**
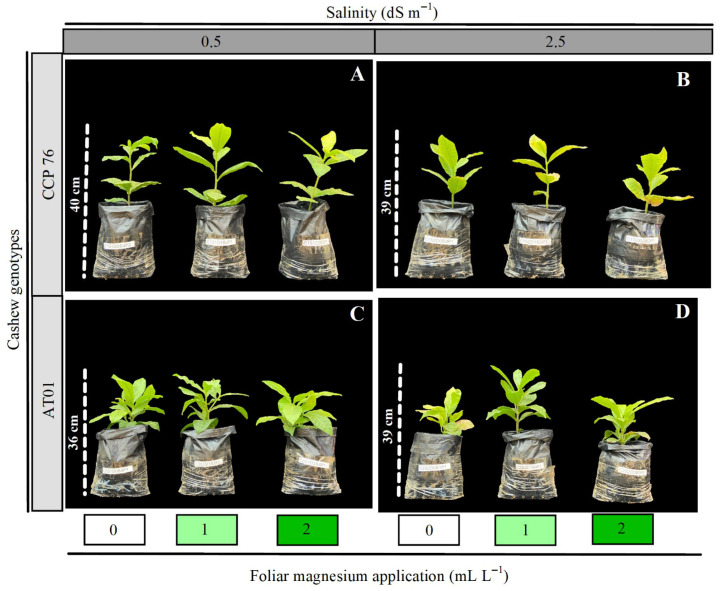
Representation of Cashew genotypes under irrigation water salinity and foliar magnesium application (**A**–**D**). Source: authors.

Significant three-way interactions among genotype, salinity, and magnesium (Mg) doses were observed for plant height (PH), number of leaves (NL), root dry mass (RDM), root-to-shoot ratio (RSR), total chlorophyll (ChlT), chlorophyll a (ChlA), chlorophyll b (ChlB), net photosynthesis (A_n_), stomatal conductance (g_s_), internal CO_2_ concentration (C_i_), and instantaneous water-use efficiency (WUE_i_) (Table 1, Table 5, and Table 6). Therefore, results are presented by analyzing: (i) genotype effects within each salinity × Mg combination, (ii) salinity effects within each genotype × Mg combination, and (iii) Mg effects within each genotype × salinity combination (Figure 1).

Within the salinity × Mg interaction, genotype effects were observed for PH, NL, RDM, and RSR (Table 1). Under non-saline conditions (0.5 dS m^−1^), CCP76 showed higher PH than AT01 at 0 and 2 mL L^−1^ Mg, whereas no significant differences between genotypes were detected at 1 mL L^−1^ Mg. Under saline conditions (2.5 dS m^−1^), genotypic differences in PH occurred only in the absence of Mg, with AT01 showing higher values than CCP76.

For NL, genotypes differed depending on salinity and Mg dose (Table 1). Under saline conditions, AT01 showed higher NL than CCP76 at 0 and 1 mL L^−1^ Mg, whereas under non-saline conditions CCP76 exhibited higher NL at 2 mL L^−1^ Mg. For RDM, genotypic differences were detected mainly under saline conditions, particularly at 2 mL L^−1^ Mg, when AT01 showed higher values than CCP76. For RSR, CCP76 exhibited higher values under non-saline conditions at all Mg doses, whereas under saline conditions AT01 showed higher RSR than CCP76, especially at 1 mL L^−1^ Mg.

Within each genotype × Mg combination, salinity significantly reduced PH, NL, and RDM in both genotypes (Table 1). In CCP76, salinity reduced RSR at all Mg doses, with a more pronounced reduction at 2 mL L^−1^ Mg. In contrast, AT01 showed increased RSR under saline conditions at 1 mL L^−1^ Mg compared with non-saline conditions.

Within each genotype × salinity combination, Mg effects on growth variables were limited and dependent on genotype (Table 1). In CCP76, Mg doses did not consistently affect PH or RDM under either salinity level, although NL increased at 2 mL L^−1^ Mg under non-saline conditions. In AT01, PH was higher at 1 mL L^−1^ Mg under non-saline conditions, whereas no consistent Mg effect was observed under saline conditions.

Shoot dry mass (SDM) showed significant interactions between genotype × salinity, genotype × Mg, and salinity × Mg (Table 2). Within the salinity × Mg interaction, AT01 showed higher SDM than CCP76 at both salinity levels, except at 1 mL L^−1^ Mg, when genotypes did not differ. Within each genotype × Mg combination, salinity significantly reduced SDM in both genotypes. No significant differences among Mg doses were detected within either genotype.

Total dry mass (TDM) was affected by genotype × salinity and genotype × Mg interactions (Table 2). AT01 showed higher TDM than CCP76 at both salinity levels. Salinity significantly reduced TDM in both genotypes. Within the genotype × Mg interaction, AT01 exhibited higher TDM at 2 mL L^−1^ Mg compared with CCP76, whereas Mg doses did not significantly affect TDM within genotypes.

Stem diameter (SD) was affected only by genotype, with AT01 showing higher values than CCP76 (Table 3). Root length (RL) was affected only by salinity, with lower values observed at 2.5 dS m^−1^ regardless of genotype or Mg dose.

ChlT, ChlA, and ChlB exhibited significant three-way interactions among genotype, salinity, and Mg dose (Table 4). Within the salinity × Mg interaction, genotypic differences were observed for all chlorophyll indices. Under non-saline conditions, AT01 showed higher ChlT than CCP76 at 0 and 2 mL L^−1^ Mg, whereas under saline conditions CCP76 showed higher ChlT at 0 and 1 mL L^−1^ Mg. At 2 mL L^−1^ Mg under saline conditions, genotypes did not differ.

Within each genotype × Mg combination, salinity reduced ChlT and ChlA in CCP76, particularly at higher Mg doses, whereas in AT01 salinity reduced ChlA only in the absence of Mg. For ChlB, salinity reduced values in AT01 in the absence of Mg, while Mg application mitigated this reduction (Table 4).

Within each genotype × salinity combination, Mg effects on chlorophyll indices were variable (Table 4). In CCP76 under non-saline conditions, Mg application reduced ChlB, whereas in AT01 under saline conditions, 1 mL L^−1^ Mg resulted in higher ChlT, ChlA, and ChlB compared with the absence of Mg.

Net photosynthesis (A_n_), stomatal conductance (g_s_), internal CO_2_ concentration (C_i_), and WUE_i_ showed significant three-way interactions among genotype, salinity, and Mg dose (Table 5). Within the salinity × Mg interaction, AT01 showed higher A_n_ than CCP76 under saline conditions at 0 and 1 mL L^−1^ Mg, whereas under non-saline conditions genotypic differences were observed only at 2 mL L^−1^ Mg.

Within each genotype × Mg combination, salinity significantly reduced A_n_ and g_s_ in both genotypes (Table 5). In AT01, g_s_ was higher at 1 mL L^−1^ Mg under saline conditions compared with the absence of Mg.

Within each genotype × salinity combination, Mg effects on gas exchange were limited and genotype-dependent. In CCP76, 1 mL L^−1^ Mg resulted in higher A_n_ compared with 0 and 2 mL L^−1^ Mg under both salinity levels. In AT01, Mg application did not consistently affect A_n_ under non-saline conditions, whereas under saline conditions 1 mL L^−1^ Mg resulted in higher g_s_.

**Table 5:** Significance of the F-test and Tukey’s test for net photosynthesis (*A_N_*, µmol CO_2_ m^−2^ s^−1^), stomatal conductance (*gs*, mol H_2_O m^−2^ s^−1^), internal CO_2_ concentration (*Ci*, µmol mol^−1^), and instantaneous water-use efficiency (*WUEi*, µmol CO_2_ m^−2^ s^−1^/mmol H_2_O m^−2^ s^−1^) of cashew seedlings under salt stress and foliar magnesium application.

Significance of F-Test						
Sources of Variation			*A_n_*	*gs*	*Ci*	*WUEi*
Block			ns	ns	ns	ns
Genotypes (G)			**	**	*	ns
Salinity (S)			**	**	ns	**
Mg			**	ns	*	ns
G × S			ns	ns	ns	ns
G × Mg			ns	ns	**	ns
S × Mg			ns	*	*	ns
G × S × Mg			*	*	**	*
CV (%)			15.20	19.54	15.92	17.4
Tukey’s Test						
Genotypes	Salinity	Magnesium	*A_n_*	*gs*	*Ci*	WUEi
CCP 76	0.5 dS m^−1^	0 mL L^−1^	16.8 αaAB	0.12 αaA	118.8 αbA	142.4 αaA
		1 mL L^−1^	18.3 αaA	0.13 αaA	133.5 αaA	138.4 αbA
		2 mL L^−1^	14.4 βaB	0.12 βaA	111.5 βaA	127.7αbA
	2.5 dS m^−1^	0 mL L^−1^	7.2 βbA	0.05 αbA	179.8 αaA	141.8 βaA
		1 mL L^−1^	7.8 βbA	0.04 βbA	107.5 αaB	177.6 αaA
		2 mL L^−1^	5.3 αbA	0.03 βbA	104.0 βaB	178.5 αaA
AT01	0.5 dS m^−1^	0 mL L^−1^	19.1 αaA	0.13 αaA	74.5 βaC	147.2 αbA
		1 mL L^−1^	20.7 αaA	0.13 αaA	117.3 αaB	161.5 αaA
		2 mL L^−1^	21.0 αaA	0.16 αaA	152.8 αaA	134.7 αaA
	2.5 dS m^−1^	0 mL L^−1^	12.2 αbA	0.06 αbAB	64.3 βaB	199.5 αaA
		1 mL L^−1^	11.2 αbA	0.08 αbA	124.8 αaA	147.3 αaB
		2 mL L^−1^	7.0 αbC	0.04 βbB	139.5 αaA	170.0 αaAB

**, * and ns correspond to significance at 1%, 5%, and non-significant, respectively. Greek letters (α and β) compare genotypes within the salinity × Mg interaction; lowercase letters (a and b) compare salinity within the genotype × Mg interaction; and uppercase letters (A, B and C) compare Mg within the genotype*salinity interaction, according to Tukey’s test at the 5% probability level.

Transpiration (E), leaf temperature (T_l_), and carboxylation efficiency (A_n_/C_i_) were affected by genotype, whereas salinity affected E, vapor pressure deficit (VPD), and T_l_ (Table 6). AT01 showed higher E, A_n_/C_i_, and T_l_ than CCP76. Increasing salinity reduced E and increased VPD regardless of genotype, while WUE was not affected by any factor.

In general, increasing salinity reduced biomass accumulation and gas exchange variables in both genotypes. The magnitude of these effects varied according to genotype and Mg dose, with significant interactions observed for most growth and physiological variables, particularly under saline conditions.

## 3. Discussion

Cashew cultivation is of major socioeconomic relevance in semiarid regions such as northeastern Brazil, where the use of saline irrigation water is increasingly common. However, cashew seedlings are particularly sensitive to salinity during early development stages. Therefore, the identification of management strategies capable of mitigating salt stress is essential. In this context, the present study demonstrates that foliar magnesium (Mg) application can partially attenuate the negative effects of salinity during early cashew development, although the magnitude and direction of the response strongly depend on the interaction among genotype, salinity level, and Mg dose.

Considering the genotype x salinity interaction, irrigation with salinity of water at 2.5 dS m^−1^ significantly reduced growth and biomass accumulation in both cashew genotypes. These reductions are primarily associated with osmotic stress caused by high salt concentration in the soil solution, which lowers water potential and restricts root water uptake [18,19]. As a consequence, cell turgor is reduced, cell expansion is inhibited, and stomatal closure is induced, limiting CO_2_ diffusion into the leaf and decreasing photosynthesis rates [2]. This physiological cascade explains the significant reduction observed in plant height, root and shoot dry mass, total dry mass, and root length in both CCP 76 and AT01, corroborating previous findings for cashew seedlings under saline conditions [8,17].

Despite these common responses, the two genotypes differed in their physiological adjustment to salinity. CCP 76 maintained total chlorophyll, chlorophyll a, and chlorophyll b indices under saline conditions, whereas AT01 exhibited significant reductions in these pigments. Nevertheless, net photosynthesis (A_n_) declined in both genotypes, indicating that photosynthetic limitation was not exclusively associated with pigment concentration. In CCP 76, the maintenance of chlorophyll levels under salinity, suggests that photosynthesis was mainly constrained by non-stomatal factors, such as reduced stomatal conductance, altered CO_2_ availability, or impairment of photosynthetic apparatus efficiency [20], rather than pigment degradation [21,22,23].

The salinity × genotype interaction was also evident in gas-exchange responses. Salinity caused marked reductions in stomatal conductance (g_s_) in both genotypes, leading to a concomitant decrease in transpiration and an increase in leaf temperature. In CCP 76, internal CO_2_ concentration (C_i_) increased under saline conditions, whereas AT01 maintained relatively stable C_i_ values, indicating distinct limitations to CO_2_ assimilation between genotypes. These results reinforce that stomatal closure is one of the earliest and most decisive physiological responses of cashew seedlings to salinity, as previously reported by Pereira et al. [18] and Carneiro et al. [9], and represents a major constraint to carbon assimilation under salt stress.

When salinity was analyzed within the genotype × Mg interaction, foliar Mg application did not consistently alleviate growth reductions caused by salt stress in CCP 76. Under high salinity, Mg application failed to increase plant height, leaf number, shoot dry mass, or total dry mass, and higher Mg doses even reduced root dry mass and the root-to-shoot ratio. These responses suggest that excessive Mg supply under saline conditions may exacerbate ionic imbalance or act as an additional stress factor, as Mg^2+^ can accumulate to toxic levels and interfere with nutrient homeostasis [14].

In contrast, AT01 exhibited a more pronounced response to Mg supplementation under saline conditions. Within the salinity × Mg interaction, the application of 1 mL L^−1^ Mg significantly increased root dry mass and root-to-shoot ratio, indicating improved biomass allocation to the root system under stress. This response suggests that AT01 has a greater capacity to use Mg to sustain root growth and maintain functional balance between shoot and root under saline conditions. Such genotypic differences are consistent with reports showing that plant responses to foliar Mg depend on genetic background, stress intensity, and nutrient interactions [24,25,26,27].

The genotype × Mg interaction under low salinity further highlighted contrasting growth strategies. CCP 76 showed increased leaf number at the highest Mg dose (2 mL L^−1^), whereas AT01 exhibited greater plant height at 1 mL L^−1^ Mg. Magnesium plays a central role in photosynthesis as the core atom of the chlorophyll molecule and as an activator of key enzymes, including ribulose-1,5-bisphosphate carboxylase/oxygenase (Rubisco), which drives CO_2_ fixation and carbohydrate synthesis [15,17,28]. In addition, Mg enhances nitrogen use efficiency and promotes sucrose transport from shoots to roots, thereby supporting root metabolism and growth [14].

The three-way interaction among genotype, salinity, and Mg was particularly evident in chlorophyll indices and gas-exchange parameters. Under saline conditions, only AT01 showed increases in chlorophyll a and chlorophyll b following application of 1 mL L^−1^ Mg, coinciding with improvements in leaf number and stomatal conductance. This indicates that Mg supplementation improved photosynthetic apparatus stability and energy production (ATP), especially in plants experiencing combined salinity and nutritional stress [15,17,28]. According to Hauer-Jákli and Tränkner [29], photosynthetic processes respond to Mg supply before visible biomass changes occur, which explains why improvements in chlorophyll indices and gas exchange were not always immediately reflected in total dry mass.

Although Mg application did not universally increase net photosynthesis under salt stress, the intermediate dose (1 mL L^−1^) enhanced photosystem performance in CCP 76 under low salinity and significantly improved stomatal conductance, internal CO_2_ concentration, and intrinsic water-use efficiency in AT01 under high salinity. These effects may be related to the role of Mg in membrane stabilization and mitigation of oxidative stress, allowing partial maintenance of stomatal opening and CO_2_ diffusion under adverse conditions [14,30,31].

Overall, this study demonstrates that the effects of foliar Mg application on cashew seedlings are strongly dependent on the interaction among genotype, salinity, and Mg dose. Salinity at 2.5 dS m^−1^ consistently reduced growth and gas-exchange performance in both genotypes, but AT01 exhibited greater physiological plasticity and tolerance to salt stress. The intermediate Mg dose (1 mL L^−1^) was the most effective in sustaining physiological function, whereas higher doses produced inconsistent or negative responses, indicating potential nutritional imbalance. These findings highlight that Mg supplementation can partially mitigate salinity stress in cashew seedlings, but its effectiveness is genotype-specific and dose-dependent, reinforcing the importance of targeted nutritional management under saline irrigation conditions.

## 4. Materials and Methods

### 4.1. Location and Plant Material

**Figure 2:**
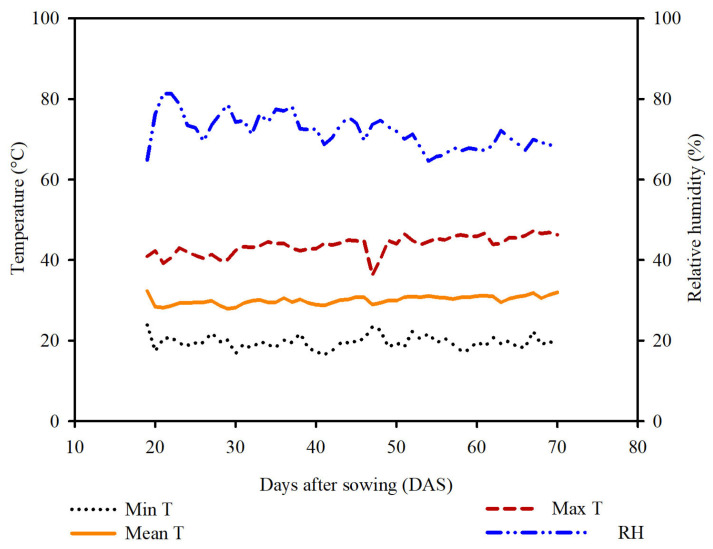
Minimum (T min), mean (T mean), and maximum (T max) temperatures, and relative air humidity inside the greenhouse during the experimental period. Source: authors.

The study was carried out in a semi-field greenhouse at the State University of Paraíba (UEPB), Campus IV, Catolé do Rocha, PB, Brazil, located at 6°20′38″ S, 37°44′48″ W, at an altitude of 275 m. The climate is classified as BSh (semi-arid, very dry, with a rainy season extending from summer to autumn) according to Köppen [32]. During the experimental period, maximum and minimum temperatures of 43.3 °C and 19.5 °C, and maximum and minimum relative humidity of 81.3% and 64.6%, respectively, were recorded (Figure 2). The study evaluated two cashew (*Anacardium occidentale* L.) genotypes subjected to magnesium application to mitigate salt stress during seedling development.

### 4.2. Experimental Design and Application Technology

The experiment was conducted in a randomized block design, arranged in a 2 × 2 × 3 factorial scheme: two cashew genotypes (G1—CCP 76 and G2—AT01), two irrigation water salinity levels (0.5 and 2.5 dS m^−1^), and three foliar magnesium doses (0, 1 and 2 mL L^−1^), with four replications of two plants each, i.e., a total of 96 plants. Foliar fertilization was performed with the commercial product FORPLANT, with a density of 1.30 g mL^−1^ and 8% Mg content. Doses were applied using a manual sprayer, divided into two applications of 7.5 mL at 35 and 50 days after sowing (DAS), totaling 15 mL per plant [33].

### 4.3. Experimental Procedures

Seeds were obtained from cashew growers in the city of Severiano Melo, RN, Brazil. Sowing was performed in 2-dm^3^ polyethylene bags, with three seeds per bag. After emergence, thinning was carried out to maintain one plant per bag.

The soil used was a Fluvent Entisol collected from an undisturbed area of the Experimental Farm at UEPB Campus IV. Samples were collected at a depth of 0–30 cm, air-dried, sieved (4 mm), and characterized for physical and chemical attributes according to EMBRAPA [34] (Table 7).

Macronutrient fertilization [35] was split into four fertigations at 30, 37, 44, and 51 DAS. In total, 50 mg N, 127 mg P_2_O_5_, 75 mg K_2_O, 29 mg Ca, 18 mg Mg, and 30 mg SO_4_^2−^ were applied per dm^3^ of soil. Nutrient sources included monoammonium phosphate (MAP), calcium nitrate, calcium chloride, magnesium sulfate, potassium sulfate, and potassium chloride. Micronutrients were supplied via foliar application of Liqui-Plex Fruit^®^ at 35 and 45 DAS, at 3 mL L^−1^, following manufacturer recommendations (Table 8).

Low-salinity irrigation water was obtained from a shallow well, with an electrical conductivity of 0.5 dS m^−1^ (Table 9). High-salinity water was prepared by adding NaCl, CaCl_2_·2H_2_O, and MgCl_2_·6H_2_O to well water, in a 7:2:1 ratio, corresponding to the predominant ionic composition of irrigation sources in Northeastern Brazil. The relationship between electrical conductivity (ECw) and concentration (mmolc L^−1^ = EC × 10) followed Rhoades et al. [36].

After soil preparation, an initial irrigation was applied to bring soil moisture close to field capacity. Subsequent irrigations were performed every two days, maintaining soil moisture near field capacity based on the drainage lysimeter method. Each irrigation included a 15% leaching fraction (LF). Applied volume was estimated using an additional plot, based on the mean water consumption of four plants, one per treatment [37]. The applied volume (Va) per plant was calculated as the difference between the previous irrigation depth (La) and the mean drainage (D), divided by the number of plants (n), as shown in equation:

(1) $$Va=\frac{La-D}{n(1-LF)}$$

The total irrigation volume applied per plant was 4.52 L, corresponding to 1.45 g of salts for plants irrigated with low-salinity water (0.5 dS m^−1^) and 7.32 g of salts for plants irrigated with saline water (2.5 dS m^−1^). At 90 DAS, an additional leaching irrigation (15%) was applied. The drained water was collected to determine drainage water electrical conductivity (ECd) and pH, using a bench conductivity meter, with EC values corrected to 25 °C and expressed in dS m^−1^. The electrical conductivity of the saturation extract (ECse) and pH (Table 10) were determined using Equation (2), as proposed by Ayers and Westcot [38] for medium-textured soils.

(2) $$ECse=\frac{ECd}{2}$$

### 4.4. Growth and Physiological Analyses

Gas exchange in cashew seedlings was evaluated at 65 DAS, between 7:00 and 10:00 a.m. Measurements were performed on fully expanded leaves from the upper third of each plant using an open-flow infrared gas analyzer (IRGA) (CIRAS-3, PP Systems, Amesbury, MA, USA), under controlled conditions of 25 °C, irradiance of 1200 µmol photons m^−2^ s^−1^, airflow of 400 mL min^−1^, and ambient CO_2_ concentration. The following parameters were recorded: net photosynthesis (A_n_, µmol m^−2^ s^−1^), transpiration (E, mmol H_2_O m^−2^ s^−1^), stomatal conductance (*gs*, mol H_2_O m^−2^ s^−1^), internal CO_2_ concentration (*Ci*, µmol mol^−1^), leaf temperature (Tf, °C), and vapor pressure deficit (VPD, kPa). From these data, the following indices were calculated: water-use efficiency (WUE = A_n_/E, µmol m^−2^ s^−1^/mmol H_2_O m^−2^ s^−1^), carboxylation efficiency (A_N_/*Ci*, decimal) [39], and intrinsic water-use efficiency (WUEi = A_N_/gs). Chlorophyll content was measured on the same leaves used for gas exchange analysis using a portable chlorophyll meter (Clorofilog CFL 1030, Falker). The device operates at three wavelengths: two in the red spectrum, near the absorption peaks of chlorophyll a and b (λ = 635 and 660 nm), and one in the near-infrared (λ = 880 nm). Results were expressed as the Falker Chlorophyll Index (FCI) [40].

After physiological analyses, seedlings were assessed for plant height (PH), stem diameter (SD), number of leaves (NL), and primary root length (RL). Plant height was measured with a graduated ruler from the soil surface to the apical meristem, and root length from the stem base to the tip of the primary root, expressed in cm. Stem diameter was measured 1 cm above the soil surface with a digital caliper, expressed in mm. The number of leaves corresponded to fully expanded green leaves per plant. Seedlings were then harvested, separated into shoot and root fractions, and oven-dried in Kraft paper bags at 65 °C with forced air circulation until constant weight. Dry mass of shoots (SDM), roots (RDM), and total biomass (TDM) was determined with an analytical balance (0.0001 g), and results were expressed in g per plant. The root-to-shoot ratio was subsequently calculated.

### 4.5. Statistical Analysis

Data were subjected to analysis of variance (ANOVA) using the F test at the 5% significance level. When significant, means were compared by Tukey’s test at 5% significance, using SISVAR^®^ 5.6 statistical software [41].

## 5. Conclusions

Salinity at 2.5 dS m^−1^ impaired cashew growth and gas exchange, with distinct responses between genotypes. Genotype AT01 exhibited greater tolerance to salt stress and higher responsiveness to foliar magnesium application, particularly at 1 mL L^−1^, which enhanced stomatal conductance, CO_2_ assimilation, and biomass accumulation. In contrast, CCP 76 showed satisfactory performance only under low salinity. The mitigating effect of magnesium is closely associated with its role in maintaining photosynthetic efficiency and ionic homeostasis. Overall, foliar Mg fertilization represents a physiologically sound strategy to improve cashew seedling performance under saline environments, provided that genotype and dosage are properly managed.

## Figures and Tables

**Table 1:** Significance of the F-Test and Tukey’s Test for plant height (PH, in cm), number of leaves (NL), root dry mass (RDM, in g), and root/shoot ratio (RSR) of cashew seedlings under saline stress and foliar magnesium application.

Significance of F-Teste						
Sources of Variation			PH	NL	RDM	RSR
Block			ns	ns	ns	ns
Genotypes (G)			**	**	*	*
Salinity (S)			**	**	**	ns
Mg			*	**	ns	ns
G × S			ns	ns	ns	ns
G × Mg			ns	**	*	**
S × Mg			ns	**	ns	**
G × S × Mg			*	**	**	**
CV (%)			11.92	12.50	18.89	15.86
Tukey test						
Genotypes	Salinity	Magnesium	PH	NL	RDM	RSR
CCP 76	0.5 dS m^−1^	0 mL L^−1^	21.2 αaA	15.7 βaB	1.28 βaA	0.53 αaA
		1 mL L^−1^	21.6 αaA	14.5 αaB	1.39 αaA	0.46 αaA
		2 mL L^−1^	22.0 αaA	21.4 αaA	1.37 αaA	0.56 αaA
	2.5 dS m^−1^	0 mL L^−1^	17.5 αbA	12.3 βbA	0.94 αbA	0.57 αaA
		1 mL L^−1^	18.8 αaA	15.8 αaA	0.75 αbAB	0.44 βaAB
		2 mL L^−1^	15.2 αbA	14.0 βbA	0.52 βbB	0.33 αbB
AT01	0.5 dS m^−1^	0 mL L^−1^	15.8 βaB	28.1 αaA	1.61 αaA	0.43 αaA
		1 mL L^−1^	19.8 αaA	14.8 αaC	1.23 αaB	0.32 βbA
		2 mL L^−1^	15.6 βaB	20.5 αaB	1.58 αaAB	0.44 βaA
	2.5 dS m^−1^	0 mL L^−1^	13.2 βaA	18.5 αbA	0.73 αbA	0.38 βaB
		1 mL L^−1^	15.9 αbA	17.1 αaA	0.90 αbA	0.62 αaA
		2 mL L^−1^	15.2 αaA	18.7 αaA	1.04 αbA	0.41 αaB

**, *, and ns correspond to significance at 1%, 5%, and not significant, respectively. Greek letters (α and β) compare genotypes within the salinity × Mg interaction; lowercase letters (a and b) compare salinity within the genotypes × Mg interaction; and uppercase letters (A, B and C) compare Mg within the genotypes × salinity interaction by Tukey’s test at the 5% probability level.

**Table 2:** Significance of F-test and Tukey’s test for shoot dry mass (SDM, in grams) and total dry mass (TDM, in grams) of cashew seedlings under salt stress and foliar magnesium application.

Significance of F-Test													
Sources of Variation								SDM					
Block								ns					
Genotypes (G)								**					
Salinity (S)								**					
Mg								ns					
G × S								**					
G × Mg								*					
S × Mg								*					
G × S × Mg								ns					
CV (%)								16.25					
Tukey’s test													
Genotypes	Salinity		SDM	Genotypes		Mg		SDM	Salinity dS m^−1^		Mg		MSPA
	dS m^−1^		g			mL L^−1^		g			mL L^−1^		g
CCP 76	0.5		2.63 βa	CCP 76		0		2.03 βA	0.5		0		3.11 aA
	2.5		1.64 βb			1		2.39 αA			1		3.47 aA
	-					2		2.00 βA			2		3.04 aA
AT01	0.5		3.78 αa	AT01		0		2.88 αA	2.5		0		1.81 bA
	2.5		1.98 αb			1		2.69 αA			1		1.59 bA
	-					2		3.08 αA			2		2.04 bA
**Significance of F-Test**													
**Sources of Variation**					**TDM**								
Block					ns								
Genotypes (G)					**								
Salinity (S)					**								
Mg					ns								
G × S					*								
G × Mg					*								
S × Mg					ns								
G × S × Mg					ns								
Tukey’s Test													
Salinity							TDM						
0.5 dS m^−1^							4.61 a						
2.5 dS m^−1^							2.62 b						
Genotypes		Salinity			TDM		Genotypes			Mg		TDM	
		dS m^−1^			g					mL L^−1^		g	
CCP 76		0.5			3.98 βa		CCP 76			0		3.15 βA	
		2.5			2.38 βb					1		3.45 αA	
		-								2		2.94 βA	
AT01		0.5			5.26 αa		AT01			0		4.05 αA	
		2.5			2.87 αb					1		3.75 αA	
		-								2		4.38 αA	

**, * and ns correspond to significance at 1%, 5%, and not significant, respectively. For the interactions: Greek letters (α and β) compare genotypes within salinity and magnesium levels; lowercase letters (a and b) compare salinity within genotypes and magnesium levels; uppercase letter (A) compare magnesium levels within salinity.

**Table 3:** Significance of the F-test and Tukey’s test for stem diameter (SD, in mm) and root length (RL, in cm) of cashew seedlings under salt stress and foliar magnesium application.

Significance of F-Test		
Sources of Variation	SD	RL
Block	ns	ns
Genotypes (G)	*	ns
Salinity (S)	ns	*
Mg	ns	ns
G × S	ns	ns
G × Mg	ns	ns
S × Mg	ns	ns
G × S × Mg	ns	ns
CV (%)	10.73	17.57
Tukey’s Test		
Genotypes	SD	RL
CCP 76	5.24 β	8.6 α
AT01	5.78 α	8.8 α
Salinity	SD	RL
0.5 dS m^−1^	5.36 a	9.5 a
2.5 dS m^−1^	5.62 a	8.0 b
Magnesium	SD	RL
0 mL L^−1^	5.5 A	8.8 A
1 mL L^−1^	5.6 A	8.5 A
2 mL L^−1^	5.4 A	8.9 A

* and ns correspond to significance at 5% and non-significant, respectively. Greek letters (α and β), lowercase letters (a and b), and uppercase letter (A) compare genotypes, salinity, and magnesium doses, respectively, according to Tukey’s test at the 5% probability level.

**Table 4:** Significance of the F-test and Tukey test for total chlorophyll (ChlT, in Falker index), chlorophyll A (ChlA), and chlorophyll B (ChlB) of cashew seedlings under salt stress and foliar magnesium application.

Significance of F-Test					
Sources of Variation			ChlT	ChlA	ChlB
Block			ns	ns	ns
Genotypes (G)			ns	**	**
Salinity (S)			**	**	**
Mg			**	**	**
G × S			**	ns	*
G × Mg			ns	**	ns
S × Mg			**	**	ns
G × S × Mg			**	**	**
CV (%)			7.45	7.97	16.13
Tukey’s test					
Genotypes	Salinity	Magnesium	ChlT	ChlA	ChlB
CCP 76	0.5 dS m^−1^	0 mL L^−1^	56.1 βaA	44.7 αaA	15.6 αaB
		1 mL L^−1^	52.5 αaA	45.7 αaA	20.6 αaA
		2 mL L^−1^	53.9 βaA	41.0 αaA	13.7 αaB
	2.5 dS m^−1^	0 mL L^−1^	58.8 αaA	43.0 αaA	15.3 αaA
		1 mL L^−1^	53.1 αaA	39.1 βbA	11.1 βbB
		2 mL L^−1^	44.6 αbB	25.0 αbB	4.9 αbC
AT01	0.5 dS m^−1^	0 mL L^−1^	66.2 αaA	47.6 αaA	16.8 αaA
		1 mL L^−1^	46.1 βbB	40.4 βaB	14.0 βaA
		2 mL L^−1^	60.3 αaA	27.5 βaC	8.3 βaB
	2.5 dS m^−1^	0 mL L^−1^	52.5 βbA	24.0 βbB	7.7 βbB
		1 mL L^−1^	55.6 αaA	43.8 αaA	14.6 αaA
		2 mL L^−1^	31.7 βbB	26.2 αaB	6.9 αaB

**, * and ns correspond to significance at 1%, 5%, and non-significant, respectively. Greek letters (α and β) compare genotypes within the salinity × Mg interaction; lowercase letters (a and b) compare salinity within the genotype × Mg interaction; and uppercase letters (A, B and C) compare Mg within the genotype*salinity interaction, according to Tukey’s test at the 5% probability level.

**Table 6:** Significance of the F-test and Tukey’s test for transpiration (E, mmol H_2_O m^−2^ s^−1^), water-use efficiency (WUE, A_n_/E, µmol CO_2_ m^−2^ s^−1^/mmol H_2_O m^−2^ s^−1^), carboxylation efficiency (A_n_/Ci, µmol CO_2_ m^−2^ s^−1^/µmol mol^−1^), leaf temperature (Tf, °C), and vapor pressure deficit (VPD, kPa) of cashew seedlings under salt stress and foliar magnesium application.

Significance of F-Test					
Sources of Variation	*E*	WUE	*A* *_n_/Ci*	Tf	V*PD*
Block	ns	ns	ns	**	**
Genotypes (G)	**	ns	*	**	ns
Salinity (S)	**	ns	ns	*	**
Mg	ns	ns	ns	ns	ns
G × S	ns	ns	ns	ns	ns
G × Mg	ns	ns	ns	ns	ns
S × Mg	ns	ns	ns	ns	ns
G × S × Mg	ns	ns	ns	ns	ns
CV (%)	21.4	16.97	83.99	1.3	4.44
**Tukey’s Test**					
Genotypes	*E*	WUE	*A* *_n_* */Ci*	Tf	V*PD*
CCP 76	2.76 β	4.37 α	0.097 β	35.9 β	3.3 α
AT01	3.34 α	4.63 α	0.188 α	36.3 α	3.2 α
Salinity	*E*	WUE	*A* *_N_* */Ci*	Tf	V*PD*
0.5 dS m^−1^	4.13 a	4.64 a	0.169 a	35.9 a	3.0 b
2.5 dS m^−1^	1.97 b	4.37 a	0.116 a	36.2 b	3.5 a

**, * and ns correspond to significance at 1%, 5%, and non-significant, respectively. Greek letters (α and β) and lowercase letters (a and b) compare genotypes and salinity, respectively, according to Tukey’s test at the 5% probability level.

**Table 7:** Chemical and physical analysis of the soil used in the experiment.

pH	OM	P	K^+^	Na^+^	Ca^2+^	Mg^2+^	Al^3+^	H + Al	CEC	V	EPS
	(%)	----(mg dm^−3^)---			----------------- (cmol_c_ dm^−3^) -------------					--- % ---	
6.5	9.7	156.8	6	1.1	5.78	0.95	0	1.2	7.44	86	1.3
CEes dS m^−1^	Ds kg dm^−3^		Sand				Silt		Clay		
			-------------------------------------- (g kg^−1^) --------------------------------------								
0.35	1.53		691.82				192.57		115.60		

OM—Organic matter.

**Table 8:** Chemical characterization of the foliar fertilizer Liqui-Plex Fruit^®^.

N	Ca	S	B	Cu	Mn	Mo	Zn	O.C.
------------------------------------------------------------g L^−1^-------------------------------------------------------								%
73.50	14.70	78.63	14.17	0.74	73.50	1.47	73.50	2.45

N—Nitrogen; Ca—Calcium; S—Sulfur; B—Boron; Cu—Copper; Mn—Manganese; Mo—Molybdenum; Zn—Zinc; O.C.—Organic carbon.

**Table 9:** Analysis of the well water used in the irrigation of cashew seedlings.

pH	EC	K^+^	Na^+^	Mg^2+^	Ca^2+^	Cl^−^	CO_3_^2−^	HCO_3_^−^	RAS
H_2_O	dS m^−1^	--------------------------mmol_c_ L^−1^------------------------------							(mmol_c_ L^−1^)^0.5^
7.21	0.50	0.32	3.20	0.64	1.15	3.91	0.29	1.36	3.38

RAS—Sodium adsorption ratio.

**Table 10:** Electrical conductivity (ECse) of the soil saturation extract under irrigation with saline water.

Salinity	Magnesium (ml L^−1^)	ECse (dS m^−1^)	
		CCP 76	AT01
0.5 dS m^−1^	0	0.61	0.59
	1	0.56	0.62
	2	0.67	0.64
2.5 dS m^−1^	0	3.16	3.05
	1	2.82	3.05
	2	3.08	3.11

## Data Availability

The original contributions presented in the study are included in the article, further inquiries can be directed to the corresponding author.
